# Rectifying Genocidal Data Stewardship: A Commentary on Ethical and Legal Obligations for Sharing Data With Tribal Entities

**DOI:** 10.2196/77946

**Published:** 2025-06-18

**Authors:** Oliver Bear Don't Walk IV, Lauren W Yowelunh McLester-Davis, Susan Brown Trinidad

**Affiliations:** 1 Department of Biomedical Informatics and Medical Education, University of Washington, Seattle, WA, United States; 2 Native American Center for Health Professions, University of Wisconsin–Madison, Madison, WI, United States; 3 Department of Bioethics and Humanities, University of Washington, Seattle, WA, United States

**Keywords:** data governance, data sharing, tribal data, Indigenous peoples, information dissemination, data science, data sovereignty, ethics, public health surveillance, privacy, deidentification, data anonymization, public health ethics, American Indian, Tribal public health

## Abstract

Access and the ability to work with Tribal data can vastly improve the ability of Tribal Nations to support their citizens’ health and well-being. In this commentary, we expand on previous calls for state and federal public health agencies to share data with Tribes by default. Previous research has described the legal and ethical lay of the land concerning public health data sharing while underscoring the importance of respect for Tribal sovereignty. In this commentary, we expand on this argument by proposing additional pathways through which data can benefit Tribes and identifying critical steps for Tribes to fully benefit from Tribal data. Specifically, we argue for (1) renewed interest and investment in Tribal data science education; (2) proactive data practices, laws, and policies that support long-term health and well-being; and (3) the federal government honoring its trust responsibility to support Tribal data resources.

## Introduction

The article “Dying in Darkness: Deviations From Data Sharing Ethics in the US Public Health System and the Data Genocide of American Indian and Alaska Native Communities” lays out a strong case for state and federal public health (PH) agencies to share data with Tribes and Tribal Epidemiology Centers (TECs) by default [1]. Schmit et al [1] expertly describe the legal and ethical lay of the land concerning PH data sharing, underscoring the need for non-Tribal PH agencies to respect Tribal sovereignty. By recognizing Tribes as legitimate PH entities, agencies can fulfill their legal obligations, ethical duties, and missions to reduce preventable deaths and harms for all people, including American Indian and Alaska Native people.

Tribal Nations have sovereign rights to “control data about their peoples, lands, and resources.” [2] These data include electronic health records, biological data, ecological (eg, nonhuman relations) data, and cultural (eg, language) data. Tribes’ use of different data types–beyond common PH data categories–has been shown to support health [3]. By cultivating the strengths that already exist within and across Tribal Nations, Tribes can improve the health and well-being for American Indian and Alaska Native peoples and Indigenous peoples globally, while also leading the field in ethical and just data science practices. We use the phrase *data science* broadly as encompassing interdisciplinary quantitative and qualitative approaches for managing data (eg, storage, access, and knowledge generation) that requires consideration for ethics, privacy, security, and downstream effects [4].

While Schmit et al [1] showed that PH data are critical for Tribal PH and that current poor stewardship of Tribal PH data by non-Tribal entities contributes to data genocide, in this commentary, we extend that discussion to consider the full range of *Tribal data*, by including any format of information and knowledge that impacts Tribal peoples, Nations, and communities [2]. To fully leverage Tribal data science and data sharing, we propose three strategies: (1) renewed interest and investment in Tribal data science education; (2) proactive data practices, laws, and policies that support long-term health and well-being; and (3) the federal government honoring its trust responsibility to support Tribal data resources.

## Promoting Tribal Data Science Through Education

Education in data science and Tribal sovereignty is paramount to truly leveraging Tribal data. Such education should value the richness of cultural knowledge and must integrate both Western and Tribal ethics and epistemologies, to combine theoretical and applied pedagogies for Tribal governance of Tribal data. Tribal and Western approaches to theory and application should be integrated while preserving the integrity of each source, for example by embracing Etuaptmumk, or Two-Eyed Seeing [5].

Similar to how the Navajo Nation institutional review board draws on Diné expertise in biology and genomics to determine appropriate research [6], Tribal Nations and TECs can combine data science expertise with Tribal knowledge and community priorities to appropriately secure, protect, and leverage Tribal data for the benefit of Tribal citizens. This expertise spans across many disciplines, as a growing number of American Indian and Alaska Native scholars have carved out paths within and outside of Western academic settings. Creating opportunities for these scholars to work within their Tribal communities will boost Tribal governance efforts.

In addition to creating job opportunities, concerted efforts to build educational opportunities across age groups on Tribal lands (eg, Native BioData Consortium’s IndigiData, Summer internship for INdigenous peoples in Genomics, Generation Data Training Course) will enable American Indian and Alaska Native students to pursue data science education, while ensuring that decisions related to this data are aligned with Tribal priorities. Tribal colleges leveraging Tribal data can (1) support training American Indian and Alaska Native scholars and future leaders with knowledge, strengths, and issues relevant to their communities; (2) provide the basis for Tribes to lead the field in developing innovative, community-responsive data science approaches, tools, and frameworks; and (3) open the possibility of Tribally-prioritized research—conducted using Tribal data, by Tribal students, with mentorship from Tribal researchers—delivering on the promise of “nothing about us, without us.”

## Proactive Data Practices, Laws, and Policies

The American Indian and Alaska Native people have long worked as data scientists and developed mechanisms for securely leveraging and sharing data with future generations in mind [7]. Tribal Nations have the opportunity to establish data practices for long-term success, by anticipating developments in a quickly evolving data landscape rather than reacting to crises as they arise. Borrowing from medical system information technology security practices, Tribal Nations can establish protocols and infrastructure to protect sensitive Tribal data before the data are even collected.

While Schmit et al [1] argued for state and federal PH agencies to share data with Tribal entities, data sharing between Tribal Nations is also key to support regional collaborations, increase sample sizes, and advance advocacy through data-driven coalition building. Discussions on widespread and automated sharing between Tribal entities should begin now. Creating spaces and regular meetings where Tribal representatives can discuss relevant values, limits, hopes, fears, lessons learned, and questions on Tribal data can precede data sharing and ensure subsequent benefits to all American Indian and Alaska Native people [8]. These conversations may draw from reflections on past experiences with data sharing between Tribes and PH entities and can later evolve to fully encompass all American Indian and Alaska Native data ecosystems.

Tribal entities have the opportunity to gain experience from Western data science efforts while effectively drawing on Tribal ethics and epistemologies, including approaches to collect, store, and transform data on health. While mainstream approaches to understanding social drivers of health are usually limited to human-controlled factors such as economic status, Tribal approaches take a more holistic view, including the health of nonhuman kin and community-level measures [9]. Tribal data sovereignty can be upheld by building data storage facilities on reservations and ensuring maximum Tribal control over data. In considering nonhuman relations and future generations, Tribes may also look to offset the environmental and social impacts of working with computers and storage hardware (eg, precious mineral mining, unethical labor, and energy intensive models) [10]. The TECs can play a pivotal role in supporting American Indian and Alaska Native data ecosystems with their capacity to facilitate aggregation, storage, and dissemination of data at the regional level, to support data governance by Tribal entities. These proactive approaches to data practices, laws, and policies will position Tribes to protect themselves while innovating on data science approaches.

## Consent, Stewardship, and Actualizing Fiduciary Responsibilities

In Nation-to-Nation agreements between the federal government and Tribes, millions of acres were provided by Tribes with the promise of returned benefits for improving the health outcomes and self-governance of American Indian and Alaska Native people. The federal government’s trust responsibility, as established in treaties and various agreements extends to the federal government’s support for Tribal Nations’ data resources. These legal and moral obligations of the federal government fundamentally support the self-determination of Tribes and data resources for the betterment of health, education, and environmental resource management. Schmit et al [1] acknowledge these legal agreements as the basis for sharing PH data by default.

Tribal governments retain sovereign authority over all PH and other data collected from their citizens, as well as data collected on their lands. Future work by Tribes can assert mandates into Tribal code requiring the sharing of any data about the Tribe, its land, and its citizens by government agencies. Individual communities and Tribes may consent to governance by placing trust in stable, transparent structures and established procedures for data access, use, protection, and enforcement.

Schmit et al [1] argue that Tribes are entitled to PH data from non-Tribal citizens, as sharing this data confers multistakeholder benefits, given that disease does not recognize borders. Increased staffing and support from Tribal entities can promote the health and well-being of local non-Tribal citizens. Additionally, collaborations and data sharing between Tribal and non-Tribal entities can produce science grounded in Tribal knowledge, with findings that support health and well-being throughout the United States.

## Conclusions

Schmit et al [1] eloquently argued that good stewardship by state and federal PH agencies requires default sharing of PH data with the Tribes. We extend this argument to consider additional ways in which these data can benefit Tribes, and the further steps needed for Tribes to derive maximum benefits from their data. As Tribes potentially consider leveraging diverse data types and rapidly advancing data science methods, they will benefit from proactively implementing laws, infrastructure, and practices, intensifying capacity-building to support data self-determination, and considering potential benefits and harms of data practices with future generations in mind.

## Abbreviations

PH: public health
TEC: Tribal Epidemiology Centers
